# The alpha-Gal syndrome: new insights into the tick-host conflict and cooperation

**DOI:** 10.1186/s13071-019-3413-z

**Published:** 2019-04-03

**Authors:** José de la Fuente, Iván Pacheco, Margarita Villar, Alejandro Cabezas-Cruz

**Affiliations:** 1 SaBio, Instituto de Investigación en Recursos Cinegéticos (IREC), Consejo Superior de Investigaciones Científicas (CSIC), Universidad de Castilla-La Mancha (UCLM)-Junta de Comunidades de Castilla-La Mancha (JCCM), Ronda de Toledo s/n, 13005 Ciudad Real, Spain; 2 Department of Veterinary Pathobiology, Center for Veterinary Health Sciences, Oklahoma State University, Stillwater, OK 74078 USA; 3 UMR BIPAR, INRA, ANSES, Ecole Nationale Vétérinaire d’Alfort, Université Paris-Est, Maisons-Alfort, 94700 France

**Keywords:** Tick, Allergy, Alpha-Gal syndrome, Vaccine, Immune response

## Abstract

This primer focuses on a recently diagnosed tick-borne allergic disease known as the alpha-Gal syndrome (AGS). Tick bites induce in humans high levels of IgE antibodies against the carbohydrate Galα1-3Galβ1-(3)4GlcNAc-R (α-Gal) present on tick salivary glycoproteins and tissues of non-catarrhine mammals, leading to the AGS in some individuals. This immune response evolved as a conflict and cooperation between ticks and human hosts including their gut microbiota. The conflict is characterized by the AGS that mediate delayed anaphylaxis to red meat consumption and certain drugs such as cetuximab, and immediate anaphylaxis to tick bites. The cooperation is supported by the capacity of anti-α-Gal IgM and IgG antibody response to protect against pathogens with α-Gal on their surface. Despite the growing diagnosis of AGS in all world continents, many questions remain to be elucidated on the tick proteins and immune mechanisms triggering this syndrome, and the protective response against pathogen infection elicited by anti-α-Gal antibodies. The answer to these questions will provide information for the evaluation of risks, diagnosis and prevention of the AGS, and the possibility of using the carbohydrate α-Gal to develop vaccines for the control of major infectious diseases.

**Electronic supplementary material:**

The online version of this article (10.1186/s13071-019-3413-z) contains supplementary material, which is available to authorized users.

## What is the alpha-Gal syndrome (AGS)?

The main objective of our research is the characterization of vector-host-pathogen molecular interactions, and translating this basic biological information into new interventions for the diagnosis, prevention and control of vector-borne diseases [1–3] (see also video at https://youtu.be/DhbBjQSuLYk). Arthropod vector-borne diseases are a growing problem worldwide, and ticks are only second to mosquitoes as vectors of human diseases and the most important vectors in animals [4–6].

The alpha-Gal syndrome (AGS) is triggered by IgE antibody response against the carbohydrate Galα1-3Galβ1-(3)4GlcNAc-R (α-Gal), which is present in glycoproteins from tick saliva and tissues of non-catarrhine mammals [7–13] (Additional file 1: Figure S1). In 2007, van Nunen et al. [7] first described the association between tick bites and the development of mammalian meat allergy. In 2009, Commins et al. [11] confirmed this association and discovered the epitope likely responsible for such allergic reactions, (α-Gal). Old World monkeys, apes and humans evolved with the inactivation of the α-1,3-galactosyltransferase (GalT) gene, which resulted in the recognition of α-Gal to produce high antibody titers against this antigen [12] (Additional file 1: Figure S1). Tick bites induce high levels of anti-α-Gal IgE antibodies in humans that mediate delayed anaphylaxis to red meat consumption, and immediate anaphylaxis to tick bites, xenotransplantation and certain drugs such as cetuximab [13, 14].

The AGS is becoming a global problem with increasing prevalence in all continents, and several tick species have been implicated in these disorders [10, 15] (Additional file 1: Figure S1). Remarkably, most of the patients that become allergic, had tolerated red meat for many years before being sensitized by tick bites [10]. This finding suggests that while IgG and IgM antibody responses to α-Gal produced by some bacteria of the gut microbiota are beneficial as they protect against infection by pathogens such as malaria parasites and tuberculosis mycobacteria, anti-α-Gal IgE antibodies induced by tick bites break the oral tolerance to food allergens and induce anaphylactic reactions to tick α-Gal-containing salivary proteins [7–15].

## Why only some individuals develop the AGS in response to tick bites?

Tick saliva is a complex mixture of pharmacologically active compounds with a role in tick attachment cement and feeding, pathogen transmission, and the inhibition of host defensive mechanisms through immunomodulatory, anti-hemostatic and anti-inflammatory molecules [16–26]. Transcriptomics, proteomics and metabolomics studies of tick salivary glands, saliva and cement discovered clusters of functionally related proteins with protease inhibitors being the most abundant group of tick salivary secreted proteins in *Ixodes scapularis* [16, 18, 20–26]. The genes coding for some of these proteins are usually expressed sequentially throughout tick feeding, bringing up the question of whether this phenomenon could be a form of antigenic variation [16]. Tick saliva modulates host immunity towards a T helper 2 (Th-2) response and suppresses inflammatory responses [27], thus deviating the host immune response to profiles that are less damaging to the feeding tick and pathogen transmission. Apart from proteins with immunomodulatory activity, ticks also produce non-protein molecules such as prostaglandin E2 (PGE_2_), which is synthesized in the tick salivary glands and secreted via the saliva into the feeding lesion [26, 28].

Humans do not synthesize the carbohydrate α-Gal, and therefore all the sources of α-Gal for the human body are from non-human origin [9, 11–13, 29]. Consequently, humans can develop a potent immune response against this carbohydrate [9, 11–13, 29]. Recently, we demonstrated that ticks synthesize α-Gal with functional GalTs with implications of this protein modification in tick feeding and *Anaplasma phagocytophilum* infection [30]. Considering these facts, evidence supports a role for α-Gal-containing tick salivary proteins in the development of the AGS, possibly in conjunction with other tick salivary components [9, 11–13, 29]. At least two possible mechanisms explain the production in humans of anti-α-Gal IgE antibodies after tick bites (Additional file 1: Figure S1). The first mechanism is supported by our current understanding of the host immune modulation by tick saliva, and proposes that α-Gal on tick salivary proteins interacts with antigen-presenting cells (APC) and B lymphocytes in the context of Th2 cell-mediated immunity induced by tick saliva. Basophils and released histamine have been implicated in IgE-mediated acquired protective immunity to tick infestations and chronic itch [31–35]. This mechanism leads to the elevation of the anti-α-Gal IgE response [16, 28]. The second mechanism needs to be demonstrated, and is based on the possibility that tick saliva contains factors that induce class switch recombination (CSR) to anti-α-Gal IgE producing B cells of pre-existing B cell clones producing anti-α-Gal IgM and/or IgG antibodies [28].

Tick salivary proteins with or without α-Gal modifications that may be involved in triggering the AGS have not been identified, but some α-Gal-containing proteins have been shown to be recognized by patients with anaphylactic reaction to tick bite and not by healthy individuals with a record of tick bites [14]. The characterization of tick proteins involved in AGS and the immune mechanisms triggering this syndrome is essential to answer the question of why only some individuals develop the AGS in response to tick bites [36–38] (Additional file 1: Figure S1). Tick sialome and alphagalactome profiles probably change as tick feeding proceeds thus highlighting the importance of the characterization of proteome changes during tick stages on the host to provide information on the abundance and risks associated with these proteins at different tick feeding stages. Furthermore, tick proteins present in the tick sialome and reacting with IgE in patients but not control sera could be used for the diagnosis of a predisposing condition for AGS. Tick sialome proteins with α-Gal modification (alphagalactome) and recognized by patients but not sera from healthy individuals exposed to tick bites could be selected as candidate protective antigens for the treatment and prevention of the AGS.

Risk factors associated to AGS may include genetic/immune mechanisms such as atopy, and ABO blood group composition leading to strong IgE response against α-Gal after tick bite, and ecological components associated to exposure to tick bites [9, 37, 39–44]. Other factors such as alcohol consumption, physical exercise, cat ownership and infection with pet-associated endoparasites, age and use of some medications may also influence the risk of developing the AGS [37, 42]. A conjunction of these and other still unknown factors may affect the development of AGS by some individuals exposed to tick bites.

## Can we benefit from the risk of developing the AGS?

Tick-host-pathogen interactions evolved as a conflict and cooperation [45]. In this context, the AGS evolved as a trade-off to benefit humans by providing immunity to pathogens containing α-Gal while increasing the risks to develop this syndrome [12, 39] (Additional file 1: Figure S1).

Some of the major infectious diseases worldwide are caused by pathogens such as *Plasmodium*, *Mycobacterium*, *Trypanosoma*, *Borrelia* and *Leishmania* species with a common characteristic of having α-Gal on their surface [39, 46–52]. As proposed for viruses with envelope-exposed α-Gal as the major evolutionary driver for the lack of functional GalT for α-Gal synthesis in humans, the possibility of developing protective antibodies against this carbohydrate resulted in an effective protection against pathogens with α-Gal [12]. This evolutionary advantage of humans relays on anti-α-Gal IgM and IgG antibodies produced in response to gut bacterial microbiota, tick infestations and/or pathogen infection with a protective effect against some infectious diseases [46–52]. However, this evolutionary cooperation between ticks and humans also leads to the conflict of increasing the risks for developing AGS in response to tick bites.

As previously proposed, we may benefit from this tick-host conflict and cooperation [46, 47] (Additional file 1: Figure S1). Gut bacteria with high α-Gal content selected from individuals with protective immune response against pathogens with α-Gal could be used to develop a probiotic-based easy to administer and low-cost vaccine that could by administered by different routes alone or in combination with α-Gal-containing tick proteins to provide protection against multiple pathogens causing major infectious diseases worldwide [46, 47]. If proven true, this would be a major advance in the control of infectious diseases affecting populations in different parts of the world.

## Conclusions

The AGS has been associated with tick bites and constitutes a growingly diagnosed disease worldwide. Nevertheless, many questions remain to be elucidated on the tick proteins and immune mechanisms triggering this syndrome, and the protective response against pathogen infection elicited by anti-α-Gal antibodies. Future research should focus at the identification of tick proteins involved in the production of anti-α-Gal IgE antibodies after tick bite, and the immune mechanisms leading to AGS. The relationship between different tick species/developmental stages and the AGS applying Koch’s postulates in GalT negative animal models would contribute to a better understanding of the disease and the evaluation of epidemiological risks. Data on blood group type should be included in epidemiological studies to better evaluate the risks for AGS associated with blood type in the population, and the putative role of anti-α-Gal IgM and IgG antibodies in protection against pathogens with α-Gal. Other factors that may affect the AGS such as endoparasite infections and microbiota composition in both humans and ticks should be considered. The answer to these questions will provide information for the evaluation of risks, diagnosis and prevention of the AGS, and the possibility of using the carbohydrate α-Gal to develop vaccines for the control of major infectious diseases.

## Additional file

**Additional file 1: Figure S1.** Poster of tick-host conflict and cooperation in the AGS: facts, challenges and possibilities.

## Abbreviations

AGS: alpha-Gal syndrome
α-Gal: Galα1-3Galβ1-(3)4GlcNAc-R
GalT: galactosyltransferase
Th-2: PGE2: T helper 2, prostaglandin E2
APC: antigen-presenting cells
CSR: class switch recombination
