# Supplementary material for: The alpha-Gal syndrome: new insights into the tick-host conflict and cooperation
Source: Parasit Vectors. 2019 Apr 3;12:154. doi: 10.1186/s13071-019-3413-z (PMC6448316; doi:10.1186/s13071-019-3413-z)
Supplement: Supplementary file 1 — Additional file 1: Figure S1. Poster of tick-host conflict and cooperation in the AGS: facts, challenges and possibilities. [file 13071_2019_3413_MOESM1_ESM.pdf]

Primers on Parasites & Vectors

The alpha-Gal syndrome: new insights into the tick-host conflict and cooperation

José de la Fuente, Iván Pacheco, Margarita Villar, Alejandro Cabezas-Cruz

Parasites & Vectors

The alpha-Gal syndrome (AGS)

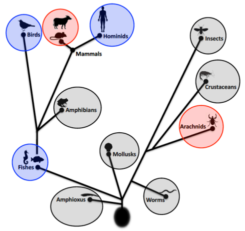

Evolutionary history of α-Gal synthesis. The capacity to synthesize α-Gal is unknown for most species (grey). However, some species synthesize α-Gal (red) while other do not have the capacity to synthesize this carbohydrate (blue).

- The α-Gal syndrome (AGS) is an allergic disease triggered by an IgE antibody (Ab) response against the carbohydrate Galα1-3Galβ1-(3)4GlcNAc-R (α-Gal), which is present in glycoproteins from tick saliva and tissues of noncatarrhine mammals. Tick bites induce high levels of anti-α-Gal IgE Abs in humans that mediate delayed anaphylaxis to red meat consumption, and immediate anaphylaxis to tick bites, xenotransplantation and certain drugs such as cetuximab [1]. Old world monkeys, apes and humans evolved with the inactivation of the α-1,3-galactosyltransferase (α1,3GalT) gene, which resulted in the recognition of α-Gal to produce high antibody Ab titers against this antigen [2].
- The evolutionary history of α-Gal synthesis has not been fully characterized, but evidence point at differences between species. Nevertheless, ticks have the galactosyltransferases (GALTs) with the capacity to synthesize this molecule with functional implications for tick biology and pathogen infection [3]. Therefore, the source of α-Gal modifications in tick salivary proteins is endogenous.

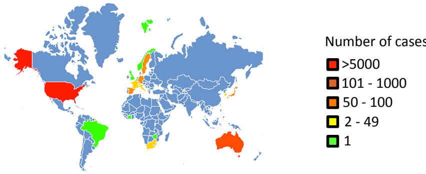

The reports of AGS cases have increased recently to cover all continents (updated after Platts-Mills TA, et al. Immunol Allergy Clin North Am. 2015; 35:247-60 .

- The the tick salivary components that are involved in the immune response mechanisms leading to the AGS have not been identified. Recently, we demonstrated that ticks produce GALTs involved in the α-Gal synthesis and developed an experimental approach for the identification and characterization of proteins with α-Gal in tick salivary glands [3,7]. Tick saliva modulates host immunity to facilitate tick feeding and possibly pathogen transmission. Therefore, the identification of tick salivary proteins with α-Gal modifications and recognized by human sera is fundamental towards understanding the immune mechanisms involved in response to α-Gal, and the treatment and prevention of the AGS before it becomes a pandemic disease.
- Tick species such as *Amblyomma americanum Ixodes ricinus* and *Ixodes holocyclus* that has been associated with the AGS in the USA, Europe and Australia, respectively constitute a good model for these studies.
- The information generated from these studies will provide a fundamental understanding of the tick-host molecular interactions and mechanisms that lead to development of the AGS. These results will also advance the possibilities for evaluating the risks of developing AGS after tick bite, and targets for the diagnosis, treatment and prevention of these allergies.

Experimental approach

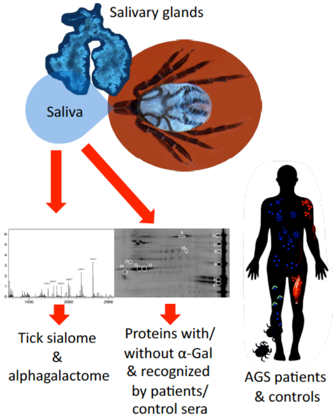

Identification of tick proteins with α-Gal and differentially recognized by individuals with AGS using proteomics approaches.

Hypotheses

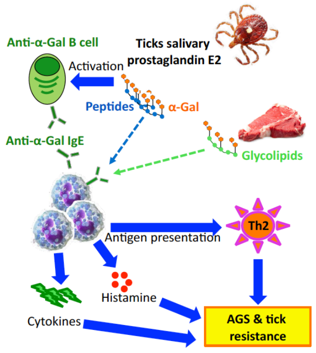

Molecular triggers of AGS (hypothesis 1 & 2).

- Hypothesis 1: Tick salivary proteins including those present in the tick cement with α-Gal modifications are involved in modulating human immune response against this carbohydrate responsible for the AGS.
- Hypothesis 2: Tick salivary prostaglandin E2 triggers antibody class switching in mature B cells, thus increasing anti-α-Gal IgE Ab levels [4].
- Hypothesis 3: Basophils and released histamine are implicated in IgE-mediated acquired protective immunity to tick infestations and chronic itch [5]. Consequently, basophil response to tick bites may be associated to α-Gal containing proteins and anti-α-Gal antibodies, possibly linking tick resistance to AGS.
- Hypothesis 4: The α-Gal producing gut bacteria can be used to develop a probiotic-based pan-vaccine to control multiple infections caused by pathogens producing α-Gal.
- Due to structural similarities between α-Gal and blood antigen B, individuals with blood type B are at lower risk of developing AGS but may be more susceptible to disease caused by pathogens with α-Gal [6].

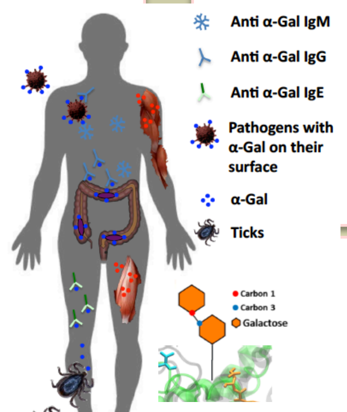

Main players in the AGS conflict & cooperation.

Conflict & cooperation

- Tick-host-pathogen interactions evolved as conflict and cooperation [8]. In this context, the AGS evolved as a trade-off to benefit humans by providing immunity to pathogens containing α-Gal while increasing the risks to develop the AGS [2,9].

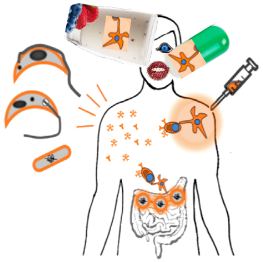

Towards a pan-vaccine for the control of major infectious diseases.

- Infectious diseases constitute a major health problem worldwide. Major infectious diseases include malaria, tuberculosis, Lyme disease and Aspergillosis caused by *Plasmodium*, *Mycobacterium*, *Borrelia* and *Aspergillus* spp., respectively. No effective vaccines exist against these pathogens. Remarkably, all these pathogens produce a common antigen, the carbohydrate α-Gal.
- Recent evidence showed that immunization with α-Gal induces a protective immune response against *Plasmodium* spp., *Trypanosoma cruzi*, and *Leishmania* spp. Humans produce anti-α-Gal Abs in response to gut microbiota bacteria producing α-Gal (reviewed by [8]). These bacteria can elicit a strong anti-α-Gal Ab response, resulting in "*sterilizing immunity*" against *Plasmodium* transmission by mosquitoes (reviewed by [9]).
- Gut bacteria with high α-Gal content selected from individuals with protective immune response against pathogens with α-Gal could be used to develop a probiotic-based easy to administer and low-cost vaccine that could by administered by different routes alone or in combination with α-Gal-containing tick proteins to provide protection against multiple pathogens causing major infectious diseases worldwide.

Sources and Further Reading:

[1] Steinke JW, Platts-Mills TA, Commins SP. The alpha-gal story: lessons learned from connecting the dots. J Allergy Clin Immunol. 2015; 135: 589-96.
[2] Galili U. Evolution in primates by "Catastrophic-selection" interplay between enveloped virus epidemics, mutated genes of enzymes synthesizing carbohydrate antigens, and natural anticarbohydrate antibodies. Am J Phys Anthropol. 2018; 168:352-63.
[3] Cabezas-Cruz A, Espinosa PJ, Alberdi P, Šimo L, Valdés JJ, Mateos-Hernández L, et al. Tick galactosyltransferases are Involved in α-Gal synthesis and play a role during *Anaplasma phagocytophilum* infection and *Ixodes scapularis* tick vector development." Sci Rep. 2018; 8: 14224.
[4] Cabezas-Cruz A, Mateos-Hernández L, Chmelař J, Villar M, de la Fuente J. Salivary prostaglandin E2: role in tick-induced allergy to red meat. Trends Parasitol. 2017; 33: 495-98.
[5] Karasuyama H, Tabakawa Y, Ohta T, Wada T, Yoshikawa S. Crucial role for basophils in acquired protective immunity to tick infestation. Front Physiol. 2018; 9: 1769.
[6] Cabezas-Cruz A, Mateos-Hernández L, Alberdi P, Villar M, Riveau G, Hermann E, et al. Effect of blood type on anti-α-Gal immunity and the incidence of infectious diseases. Exp Mol Med. 2017; 49:e301.
[7] Mateos-Hernández L, Villar M, Moral A, García Rodríguez C, Alfaya Arias T, De La Osa V, et al. Tick-host conflict: immunoglobulin E antibodies to tick proteins in patients with anaphylaxis to tick bite. Oncotarget 2017; 8; 20630-44. (13).
[8] de la Fuente J, Villar M, Cabezas-Cruz A, Estrada-Peña A, Ayllón N, Alberdi P. Tick-host-pathogen interactions: conflict and cooperation. PLoS Pathog. 2016; 12:e1005488.
[9] Cabezas-Cruz A, de la Fuente J. Immunity to α-Gal: toward a single-antigen pan-vaccine to control major infectious diseases. ACS Cent Sci. 2017; 3: 1140-42.
